# A Novel Role for C5a in B-1 Cell Homeostasis

**DOI:** 10.3389/fimmu.2018.00258

**Published:** 2018-02-19

**Authors:** Katharina Bröker, Julia Figge, Albert F. Magnusen, Rudolf A. Manz, Jörg Köhl, Christian M. Karsten

**Affiliations:** ^1^Brandenburg Medical School, University Hospital Brandenburg, Center of Internal Medicine II, Brandenburg a. d. Havel, Germany; ^2^Institute for Systemic Inflammation Research, University of Lübeck, Lübeck, Germany; ^3^Division of Immunobiology, Cincinnati Children’s Hospital Medical Center, Cincinnati, OH, United States

**Keywords:** complement, B-1 cells, natural antibodies, C5a, C5, CXCL13

## Abstract

B-1 cells constitute a unique subpopulation of lymphocytes residing mainly in body cavities like the peritoneal cavity (PerC) but are also found in spleen and bone marrow (BM). As innate-like B cells, they mediate first line immune defense through low-affinity natural IgM (nIgM) antibodies. PerC B-1 cells can egress to the spleen and differentiate into nIgM antibody-secreting plasma cells that recognize conserved exogenous and endogenous cellular structures. Homing to and homeostasis within the PerC are regulated by the chemokine CXCL13 released by PerC macrophages and stroma cells. However, the exact mechanisms underlying the regulation of CXCL13 and B-1 homeostasis are not fully explored. B-1 cells play important roles in the inflammatory response to infection, autoimmunity, ischemia/reperfusion injury, obesity, and atherosclerosis. Remarkably, this list of inflammatory entities has a strong overlap with diseases that are regulated by complement suggesting a link between B-1 cells and the complement system. Interestingly, up to now, no data exist regarding the role of complement in B-1 cell biology. Here, we demonstrate for the first time that C5a regulates B-1 cell steady-state dynamics within the peritoneum, the spleen, and the BM. We found decreased B-1a cell numbers in the peritoneum and the spleen of C5aR1^−/−^ mice associated with increased B1-a and B1-b numbers in the spleen and high serum titers of nIgM antibodies directed against phosphorylcholine and several pneumococcal polysaccharides. Similarly, peritoneal B-1a cells were decreased in the peritoneum and splenic B-1a and B-1b cells were increased in C5aR2^−/−^ mice. The decrease in peritoneal B-1 cell numbers was associated with decreased peritoneal CXCL13 levels in C5aR1^−/−^ and C5aR2^−/−^ mice. In search for mechanisms, we found that combined TLR2 and IL-10 receptor activation in PerC macrophages induced strong CXCL13 production, which was significantly reduced in cells from C5aR1- and C5aR2-deficient mice and after combined C5aR-targeting. Such stimulation also induced marked local C5 production by PerC macrophages and C5a generation. Importantly, peritoneal *in vivo* administration of C5a increased CXCL13 production. Taken together, our findings suggest that local non-canonical C5 activation in PerC macrophages fuels CXCL13 production as a novel mechanism to control B-1 cell homeostasis.

## Introduction

B-1 lymphocytes are innate-like B cells that mediate first broad and unspecific, antibody-based immune responses as well as long-lasting T cell-independent (TI) protective immunity against infections (1, 2). Most B-1 cells reside in body cavities such as the peritoneum or the pleura. Further, they can be found in lower numbers in spleen and bone marrow (BM) (3–6) where they spontaneously secrete high levels of natural IgM (nIgM) antibodies (6, 7). Phenotypically, B-1 cells are characterized as CD45R^lo^, surface IgM^hi^, sIgD^lo^, CD19^hi^, and CD43^+^ cells. They can be divided into CD5^+^ B-1a cells and CD5^–^ B-1b cells (1). Further, some peritoneal cavity (PerC) B-1 cells are positive for the αM integrin (CD11b), which defines sequential stages of B-1 cells (8).

B-1a cells serve as the main source of low affinity, polyreactive nIgM antibodies against conserved exogenous and endogenous structures like the bacterial antigen phosphorylcholine (PC) (9, 10), which are present in the circulation even without previous antigen exposure (11). B-1b cells produce antibodies in a TI manner, e.g., against LPS or other polysaccharides from encapsulated pathogens (12). In line with these functional properties, B-1 cells are critical for the early control of infections with encapsulated bacteria like *Streptococcus pneumoniae* (12), mediate protection from bacterial infection with *Borrelia hermsii* (13), viral infection with *Influenza* (14) or fungi (15). Further, B-1 cell-derived nIgM antibodies exhibit a protective role in atherosclerosis by mediating clearance of altered self-antigens (16–18). In addition, several findings suggest a role of B-1 cells in autoimmune diseases including type I diabetes (19) or systemic lupus erythematosus (20) through interaction with other cell types. Despite their importance in warding off pathogens, controlling autoimmune diseases and atherosclerosis, the exact mechanisms regulating B-1 cell homeostasis are still ill-defined.

Previous findings suggest that B-1 cell homing to body cavities is strongly dependent on CXCL13 (21). Lymphocyte-rich follicles express high levels of this chemokine, which directs circulating CXCR5^+^ B-1 cells to the PerC. Consequently, mice lacking CXCL13 have a substantially reduced pool of peritoneal and pleural B-1a and B-1b cells. On the other hand, stimulation with exogenous cytokines such as IL-10 and IL-5 (22) or TLR ligands (4, 5, 23) as well as infection with *S. pneumoniae* (24) or the *Influenza* virus (25) promote trafficking of body cavity B-1 cells to secondary lymphoid organs and mucosal sites.

First-line host defense includes the recognition of pathogens by several pattern recognition receptors (PRRs). These PRRs sense potential threats that compromise the integrity of host cells, tissues, or even the entire body. They can either be membrane-bound, like TLRs, or soluble. The latter include C1q and mannan-binding lectins of the complement system, among others (26). Upon target binding, they activate the system through a sequence of proteolytic events eventually resulting in multiple cleavage fragments that either fuel the cascade or bind to specific complement receptors on a variety of innate or adaptive immune cells (27). The C3b cleavage fragment and derivatives thereof serve as opsonins to facilitate phagocytosis of microbes. In contrast, C3a and the small cleavage fragment of C5, C5a bind to their cognate C3aR, C5aR1, and C5aR2 and exert pro-inflammatory and many immunoregulatory functions [reviewed in Ref. (28)]. Both receptors for C5a, i.e., C5aR1 and C5aR2 are expressed on several innate immune cells including neutrophils, macrophages, dendritic cells, and on non-immune cells (29–31). In addition to the canonical generation by the classical, lectin, or alternative pathway, C3a and C5a may also be produced locally by cell-derived proteases (32). Many exogenous and endogenous structures such as LPS, glycolipids, phosphatidylserine, and modified LDL are recognized by both, complement-derived danger sensors and TLRs, suggesting that complement receptor pathways may intersect with TLR pathways. Indeed, cross talk between TLRs and C5aR1 regulates the development of Th1/Th2/Th17 and regulatory T cell responses critical for infection with intracellular parasites (33, 34), autoimmune diseases (34–37), and allergic asthma (38, 39). The expression of C5aRs on B-1 cells and the impact of a potential cross talk between TLRs and complement receptors on B-1 cell immunity has not been investigated yet.

Here, we specifically addressed regulatory effects of the anaphylatoxin C5a and its receptors on B-1 cell biology. We found that C5a controls B-1 cell homeostasis in the PerC, spleen, and BM. B-1a cell numbers in the PerC were significantly decreased in C5aR1- and C5aR2-deficient mice, which was associated, at least in C5aR1^−/−^ mice, with decreased CXCL13 levels. Further, B-1b cells were lower in the BM of C5aR1^−/−^ mice. This decrease in B-1 cells in the PerC and the BM of C5aR-deficient mice was associated with increased B-1 cell numbers in the spleen. Importantly, C5aR1-deficient mice exhibit elevated levels of nIgM antibodies reactive with *S. pneumoniae* antigens. Mechanistically, we uncovered that peritoneal macrophages produce C5 and cleave it into C5a by a cell-derived protease in response to IL-10 and TLR2 ligation. Such C5a is required to drive CXCL13 production by peritoneal macrophages, thereby contributing to B-1 cell homeostasis in the PerC. In line with this view, we found that i.p. injection of C5a increased peritoneal CXCL13 levels. Thus, our findings demonstrate a novel role for C5a and its receptors in the regulation of B-1 cell biology under steady-state conditions.

## Materials and Methods

### Reagents

The monoclonal BV421-labeled Ab against CD43 (S7) was purchased from BD Biosciences; AF700-labeled Ab against CD11b (M1/70), unlabeled Ab against CD16/32 (Fc-Block, 93), eF450-labeled Ab against CD24 (M1/69), APC-labeled Ab against CD45R/B220 (RA3-6B2), PerCP-Cy–Cy5.5-labeled Ab against CD5 (53–7.3.), PE-labeled Ab against CD5 (53–7.3.) as well as PE-Cy7–labeled Ab against IgM (II/41) were purchased from eBioscience (Affymetrix). Further, APC-labeled Ab against CD19 (6D5) and C5aR1/CD88 (20/70), PB-labeled Ab against CD23 (B3B4), FITC-labeled Ab against CD43 (S11), and AF700-labeled Ab against IgD (11-26c.2a) were purchased from BioLegend. The C5-specific Ab (BB5.1) was purchased from Hycult Biotech and labeled with AF647 using kit A20186 from Thermo Fisher Scientific.

Red blood cell lysis (RBCL) buffer was prepared using 155 mM NH_4_Cl, 10 mM KHCO_3_, and 0.1 mM EDTA (all from Sigma-Aldrich). DMEM, PBS, HEPES, l-glutamine, penicillin, and streptomycin were from Life Technologies. FCS was from PAA. Pam3CSK4 was purchased from Invivogen, IL-10 from R&D Systems, and human C5 from Complement Technologies. The C5aR antagonist (A8^Δ71–73^) was generated as described previously (40). The Stat-3 inhibitor Stattic was from Calbiochem/Merck. Cytofix/Cytoperm was purchased from BD Biosciences, Fluoromount-G from Southern Biotech, and DAPI from Life Technologies. ELISA Kits for detection of CXCL12, CXCL13, and IL-10 were purchased from R&D Systems. 1-Step™ Ultra TMB-ELISA substrate was purchased from Thermo Scientific. Goat anti-mouse IgM and IgM-horseradish peroxidase (HRP) were from Southern Biotech. BSA and Tween^®^ 20 were from Sigma-Aldrich. PC was from Biosearch Technologies, pneumococcal polysaccharides (PP) were purchased from ATCC. For SDS PAGE and Western blot analysis, we used Mini-Protean TGX Precast gels (4–15%), Precision^+^ Western C Standard, Streptactin-HRP, all purchased from Bio-Rad. Streptavidin-HRP was from R&D Systems and milk powder from Rockland. Human C5a was from Complement Technologies. Biotin-conjugated mAb 557 against C5a/C5 was from Hycult Biotech. DNase I for mRNA generation was from Fermentas. Primers for real-time RT-PCR, and all other reagents for RT-PCR were from ThermoFisher.

### Animals

Wild-type (wt) C57BL/6J mice were purchased from JANVIER LABS. Wt BALB/c mice were purchased from Charles River. C5aR1^−/−^, C5aR2^−/−^, floxed GFP-C5aR1 knock-in (30), and C5^−/−^ as well as MyD88^−/−^ and TLR2^−/−^ mice on the C57BL/6J background and C5aR1^−/−^ and C5aR2^−/−^ mice on the BALB/c background were bred and maintained in an SPF animal facility of the University of Lübeck. The C5^−/−^ mice originate from B10.D27nSnJ mice and have been backcrossed in the Lambris laboratory for 10 generations on the C57BL/6J background. IL 10-reporter (VertX mice) and B-cell-specific IL-10^−/−^ mice (and corresponding litter mate controls) were kindly provided by C. Karp (41). All animals were used at 8–12 weeks of age and handled in accordance with the appropriate institutional and national guidelines. All animal studies were reviewed and approved [number: V242—81505/2016 (19-2/17)] by local authorities of the Animal Care and Use Committee (Ministerium für Energiewende, Landwirtschaft, Umwelt, Natur und Digitalisierung, Kiel, Germany) and performed by certified personnel. Both, male and female mice were equally used for the experiments.

### Preparation of Serum, Peritoneal, Splenic, and BM Cells

Blood was taken by puncture of the submandibular vein and was directly collected in serum-separating tubes (BD). All mice were killed by cervical dislocation under anesthesia before organ removal. For isolation of peritoneal cells, the PerC was flushed with 5 ml of ice-cold PBS. For BM preparation, femurs, tibias, and humeri were removed and flushed with PBS. Splenic cells were isolated by mechanical disruption. Single cell suspensions were obtained by pressing the cells through a cell strainer (40 µm nylon, BD) using the plunger of a 5 ml syringe. Cell strainers were then flushed three times with PBS. If necessary, RBCs were removed by incubating the cells in RBCL buffer for 3 min and washing in PBS to stop lysis.

### Flow Cytometry

Phenotypic characterization of the cells was performed using a LSRII cytometer or an Aria III cytometer (both BD). Cells were incubated in PBS 1% BSA with Fc block (anti-CD16/32) for 15 min at 4°C and subsequently stained with the corresponding antibodies as outlined below for 15 min at 4°C and then washed with PBS. Staining of peritoneal cells was performed with anti-CD11b-AF700, anti-CD45R-APC, anti-CD5-PE or anti-CD5-PerCPCy5.5, anti-CD43-BV421, anti-IgM-PECy7 antibodies. Anti-CD19-APC, anti-CD23-Pacific Blue, anti-CD43-BV421, anti-IgD-AF700, anti-IgM-PECy7, and anti-CD5-PE antibodies were used for staining of splenic cells. BM cells were stained with anti-CD19-APC, anti-IgM-PeCy7, anti-CD24-Pacific Blue, anti-CD43-FITC, and anti-CD5-PE antibodies.

### Culture and Stimulation of Peritoneal Cells

For phenotypical characterization of peritoneal macrophages, PerC cells were resuspended in complete medium (DMEM supplemented with 10% heat-inactivated FCS, 1% l-glutamine, 100 IU/ml penicillin, 100 µg/ml streptomycin, 10 mM HEPES), transferred into a 24-well-plate (0.6 × 10^6^/ml) and incubated overnight to separate adherent PerC cells. Non-adherent PerC cells were then collected and adherent PerC cells were washed twice with PBS. Supernatants (SNs) from these washing steps were collected together with non-adherent PerC cells. Adherent PerC cells were supplemented with 250 µl of fresh medium; non-adherent PerC cells were transferred to a 96-well-plate (0.5 × 10^6^/ml). Adherent PerC cells were stimulated with Pam3CSK4 (40 ng/ml, Invivogen) and/or IL-10 (4 ng/ml, R&D Systems) in the presence or absence of the C5aR antagonist A8^Δ71–73^ [5 µM (40)]. Non-adherent PerC cells were stimulated with Pam3CSK4 (400 ng/ml, Invivogen). Cells were incubated for 24 h, and cell culture SNs were collected for further analysis. In some experiments, Stat-3 signaling in adherent PerC cells was blocked before TLR2 stimulation using the Stat-3 inhibitor Stattic (50 µM, 60 min, Calbiochem/Merck). Incubation of cells with DMSO served as negative control. In experiments, where we determined exogenous production of C5 in peritoneal macrophages, adherent PerC cells were cultured in serum-free medium (DMEM, 1% l-glutamine, 100 IU/ml penicillin, 100 µg/ml streptomycin, 10 mM HEPES).

### Determination of Peritoneal Chemokine and Cytokine Production

IL-10, CXCL12, and CXCL13 levels in peritoneal lavage fluid as well as in cell culture SNs were determined using Duo Set ELISA Kits (R&D systems) following the manufacturer’s protocol. In some experiments, mice were injected i.p. with 200 nM C5a (Hycult Biotech) in PBS 6 h before peritoneal lavage and analysis of peritoneal CXCL13 levels.

### Assessment of *In Vivo* Antibody Production

To determine serum levels of total as well as specific nIgM antibodies for PC and PP of different serotypes (PP1, PP2, PP4, PP51), plates were coated with 5 µg/ml of the respective antigen overnight at 4°C. Plates were blocked with PBS 1% BSA for 1 h and then incubated with sera for another hour (both at 37°C). IgM antibodies were detected using HRP-conjugated goat anti-mouse IgM specific antibodies (1:5,000, Southern Biotech), which were incubated for 1 h at 37°C and substrate reagent. All washing steps were performed with PBS/0.05% Tween.

### Intracellular C5 Staining

To determine intracellular expression of C5 in peritoneal macrophages, total peritoneal cells from wt and MyD88^−/−^ mice were incubated in six-well chamber slides (Sarstedt). Adherent cells were separated and stimulated as described above. Cells were then washed three times with PBS and permeabilized by incubation in Cytofix/Cytoperm buffer (BD) for a minimum of 30 min. Cells were stained with DAPI and the anti-C5 antibody (BB5.1) in Perm/Wash buffer (BD) for 15 min, washed three times with PBS and mounted with Fluoromount-G (Southern Biotech). C5 expression was analyzed using an Olympus FV 1000 confocal microscope and the Imaris 8.0 software.

### RNA Isolation and Real-time PCR

To determine C5 mRNA expression, peritoneal macrophages were isolated and stimulated as described above and RNA isolation was performed using the RNeasy Kit (Qiagen) according to the manufacturer’s instructions. Primers, TaqMan probes, and TaqMan assay reagents for murine C5 (Mm00439275_m1) and GAPDH (Mm99999915_g1) were purchased from Thermo Fisher. The RNA was diluted to 5 ng/µl, and contaminating DNA was removed by DNA digestion (DNAfree™, Ambion). C5 and GAPDH real-time RT-PCR assay were performed using TaqMan One Step RT-PCR Master Mix reagent plus forward primer, reverse primer, C5 and GAPDH TaqMan probe (total 10 pmol), and 50 ng RNA. Amplification and detection were performed using a CFX96 Real Time System (Bio-Rad) with the following profile: 25°C for 2 min, then, 53°C for 10 min, and 95°C for 2 min, followed by 45 cycles at 95°C for 15 s, and 60°C for 30 s. Results were based on relative quantification with the comparator CT method. For final examination, the target group (unstimulated cells) was set as reference with a value of 1. The treated test group was set as being *x*-fold difference relative to the reference.

### C5 Cleavage Assay and Western Blot for C5a

Peritoneal cells from wt C57BL/6J mice (6 × 10^6^/ml) were incubated overnight in adherent 24-well-plates in complete medium without FCS (DMEM supplemented with 1% l-glutamine, 100 IU/ml penicillin, 100 µg/ml streptomycin, 10 mM HEPES) in order to separate adherent and non-adherent cells. Adherent cells were washed twice and supplemented with 250 µl of fresh medium without FCS. Cells were stimulated with Pam3CSK4 (40 ng/ml, Invivogen) and IL-10 (4 ng/ml, R&D Systems) and incubated for 4 h in the presence or absence of C5 (60 µg, Complement Technology). Cell culture SNs were collected and used for Western Blot analysis of C5a production. Cell culture SNs were separated by SDS-PAGE according to standard procedures using Mini-Protean TGX Precast gels (4–15%) (Bio-Rad). Protein was subsequently transferred onto a Trans-Blot nitrocellulose membrane using a Trans-Blot SD system (both from Bio-Rad). Western Blot analysis was then performed according to standard procedures. Briefly, the membrane was incubated with a biotinylated C5/C5a-specific antibody (Hycult Biotech, clone 557; 4 µg/ml) in TBS + Tween + milk powder for 2 h at room temperature. After washing, the membrane was incubated with streptavidin HRP (R&D Systems) in TBS + Tween + milk powder for 2 h at room temperature. Detection was performed using the Immun Star WesternC Kit (Bio-Rad) and Fusion SL (Vilber Lourmat).

### Statistical Analysis

**Figure 1:**
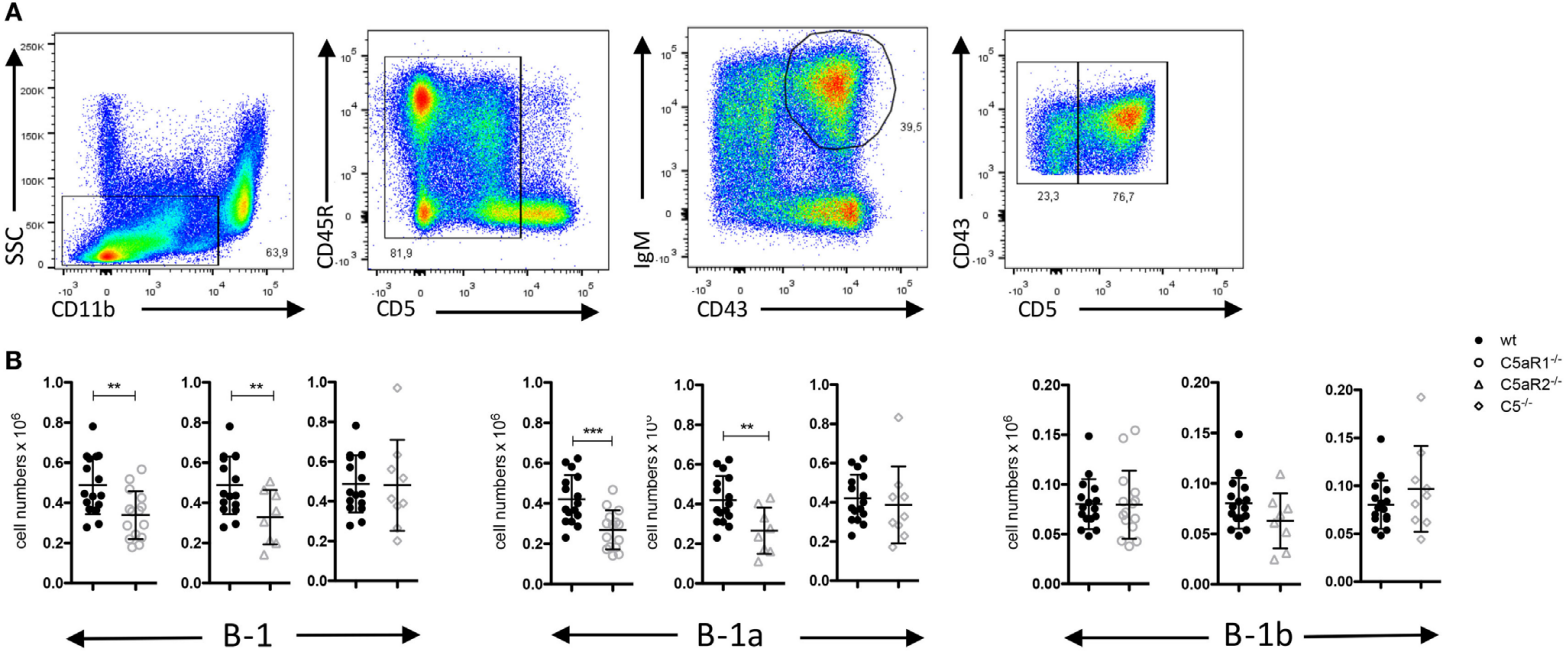
B-1 cell numbers in the peritoneal cavity (PerC) of wild-type (wt), C5aR1^−/−^, C5aR2^−/−^, and C5^−/−^ mice. **(A)** Gating strategy to identify total B-1, B-1a and B-1b cells in the PerC. **(B)** Total B-1, B-1a and B-1b cell numbers in the peritoneum of wt, C5aR1^−/−^, C5aR2^−/−^, and C5^−/−^ mice (all on C57BL/6J background). Values shown are the mean ± SD. Statistical differences between wt and C5- or C5aR-deficient mice were determined by unpaired *t*-test. **p < 0.01, ***p < 0.001.

**Figure 2:**
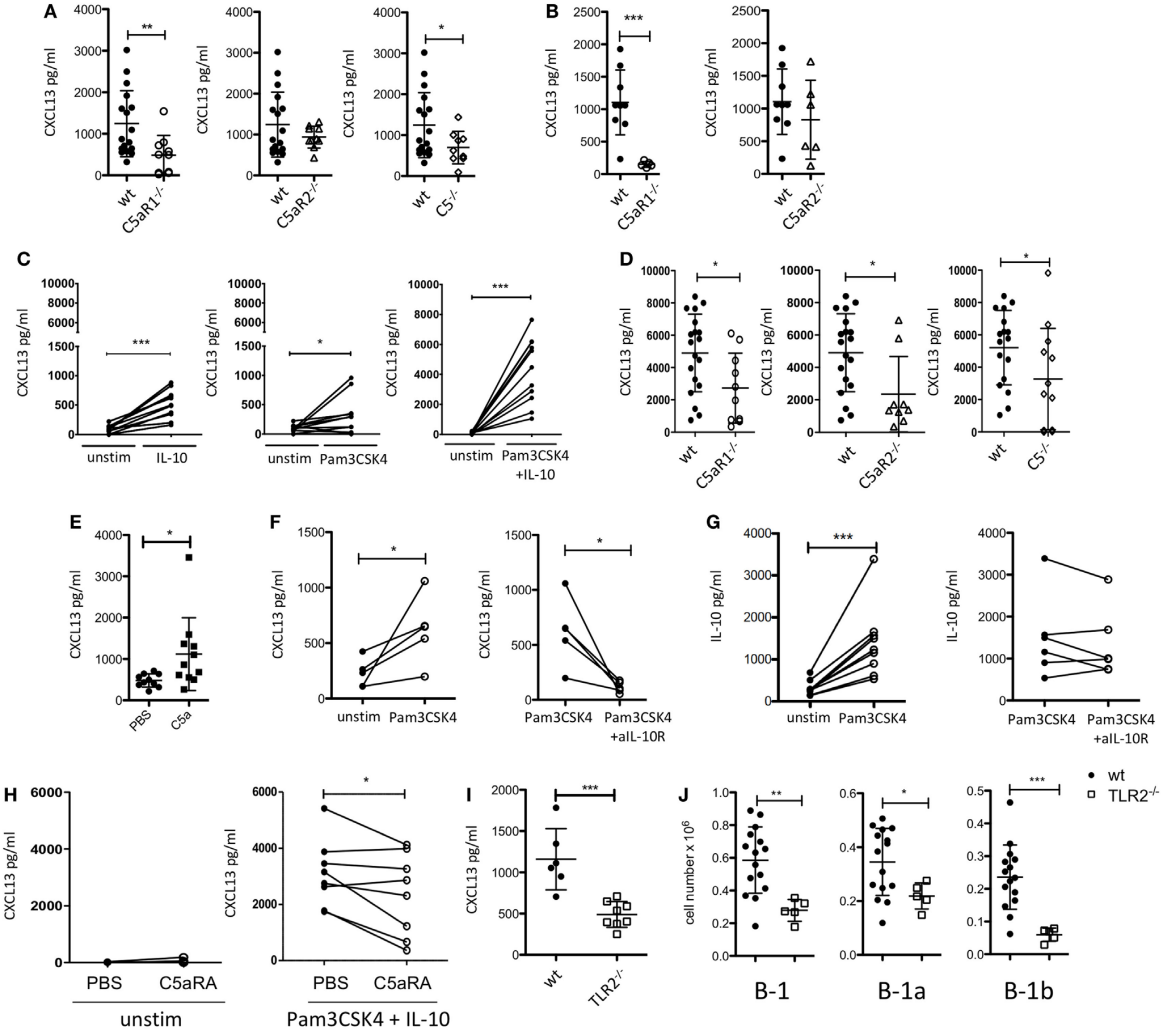
Impact of the C5/C5a/C5aR/TLR2 axes on peritoneal CXCL13 production under steady-state and inflammatory conditions. **(A)** CXCL13 concentrations in the peritoneal lavage fluid of wild-type (wt), C5aR1-, C5aR2-, and C5-deficient mice (C57BL/6J background) under steady-state conditions. **(B)** CXCL13 concentrations in the peritoneal lavage fluid of wt, C5aR1- and C5aR2-deficient mice (BALB/c background) under steady-state conditions. **(C)** *In vitro* CXCL13 production by peritoneal macrophages from wt mice (C57BL/6J background) 24 h after stimulation with IL-10, Pam3CSK4, or Pam3CSK4 + IL-10. **(D)** CXCL13 production from wt, C5aR1^−/−^, C5aR2^−/−^, or C5^−/−^ peritoneal macrophages (all on C57BL/6J background) in response to Pam3CSK4 + IL-10. **(E)** CXCL13 concentrations in the peritoneal lavage fluid of wt mice (C57BL/6J background) 6 h after i.p. injection with 200 nM C5a or PBS. **(F,G)** *In vitro* CXCL13 **(F)** or IL-10 **(G)** production by total peritoneal cavity (PerC) cells from wt mice 24 h after stimulation with Pam3CSK4 compared to unstimulated (left panel) or Pam3CSK4-stimulated cells compared to simultaneous stimulation of Pam3CSK4 together with +anti-IL-10R Ab (right panel). **(H)** CXCL13 production from unstimulated (left panel) or Pam3CSK4 + IL-10-stimulated (right panel) adherent PerC cells from wt mice in the presence or absence of C5aRA (5 µM). **(I)** CXCL13 concentrations in the peritoneal lavage fluid of wt and TLR2-deficient mice (C57BL/6J background) under steady-state conditions (left). **(J)** Total B-1, B-1a, and B-1b cell numbers in the peritoneum of TLR2-deficient as compared to wt mice (both C57BL/6J background). Values shown are the mean ± SD **(A,B,D,E,I,J)**. Statistical differences between wt and knockout mice were determined using an unpaired *t*-test **(A,B,I,J)** or Mann–Whitney test **(D)**; statistical differences between different stimulation conditions were determined using an unpaired **(E)** or paired *t*-test **(C,F–H)**. *p < 0.05, **p < 0.01, ***p < 0.001.

**Figure 3:**
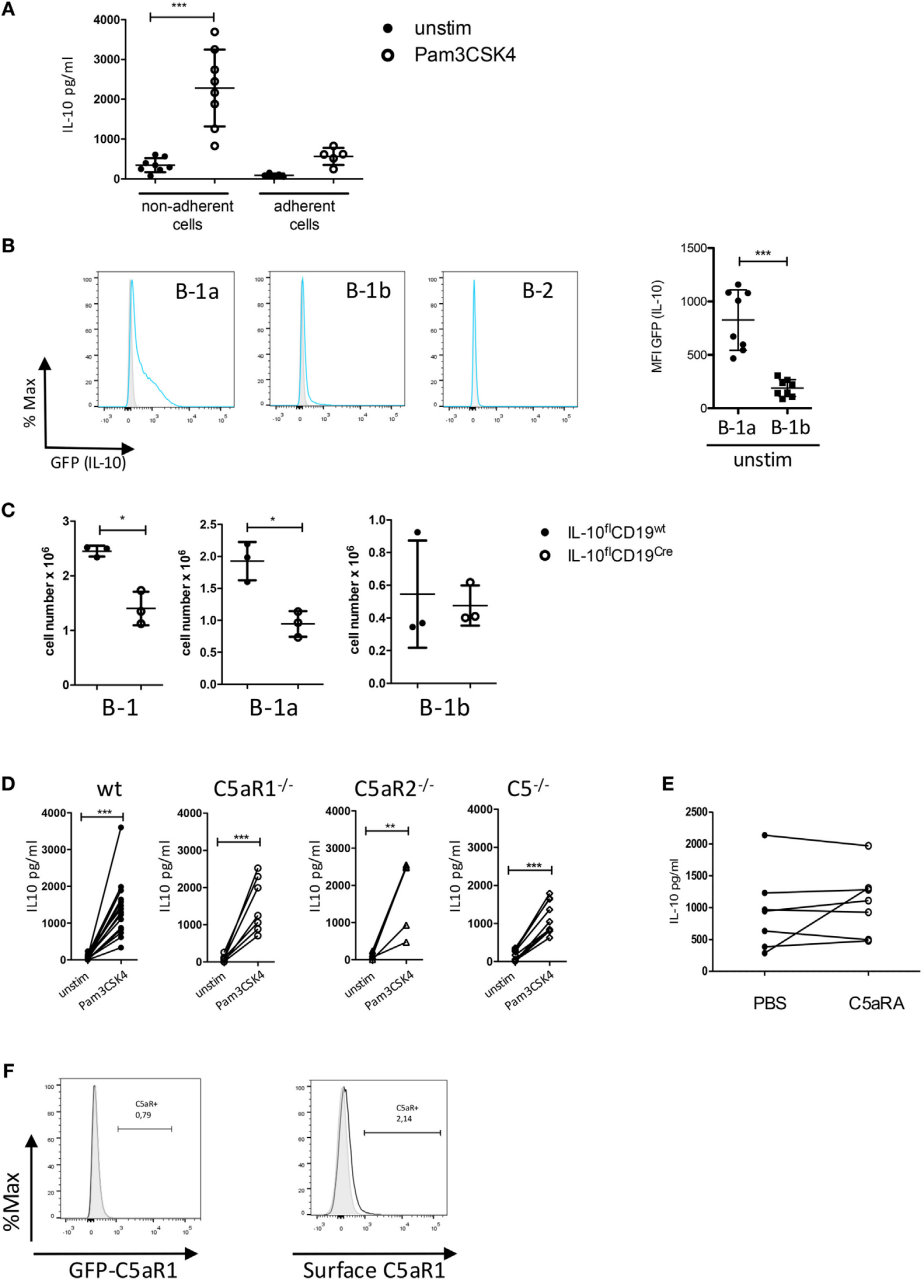
Impact of the C5/C5a/C5aR axes on IL-10 production from peritoneal B-1 cells under steady-state and inflammatory conditions. **(A)** IL-10 production from non-adherent and adherent PerC cells in response to Pam3CSK4. **(B)** Determination of IL-10 production in peritoneal B-1a, B-1b, and B-2 cells using GFP-IL-10 knockin mice (Vert-X) (grey histogram = wildtype control, blue line = unstimulated Vert-X mouse). The right panel shows the quantification of IL-10 production (shown as MFI of the GFP signal) from B-1a and B-1b cells. **(C)** Impact of IL-10 deletion in CD19^+^ B cells on the number of peritoneal B-1 (left), B-1a (middle), and B-1b (right) cells under steady-state conditions. **(D)** Impact of C5, C5aR1, or C5aR2 deficiency on IL-10 production from non-adherent PerC cells in response to Pam3CSK4 stimulation. **(E)** Impact of C5aR1/2 blockade (using the C5aRA A8^Δ71–73^) on Pam3CSK4-induced IL-10 production. **(F)** GFP-C5aR1 expression (left panel) or C5aR1 surface expression (right panel) in peritoneal B-1 cells. Values shown in **(A–C)** are the mean ± SD. Statistical differences between non-adherent and adherent PerC cells with or without stimulation were determined using Kruskal–Wallis test with Dunn’s *post hoc* test **(A)**. Statistical differences between MFI expression in B-1a and B-1b cells were determined using unpaired *t*-test **(B)**; statistical differences in B-1 cell numbers between control mice and B cell-specific IL-10 knockout mice were determined using Mann–Whitney test **(C)**; statistical differences between different stimulation conditions were determined using a paired *t*-test **(D,E)**. *p < 0.05, **p < 0.01, ***p < 0.001.

**Figure 4:**
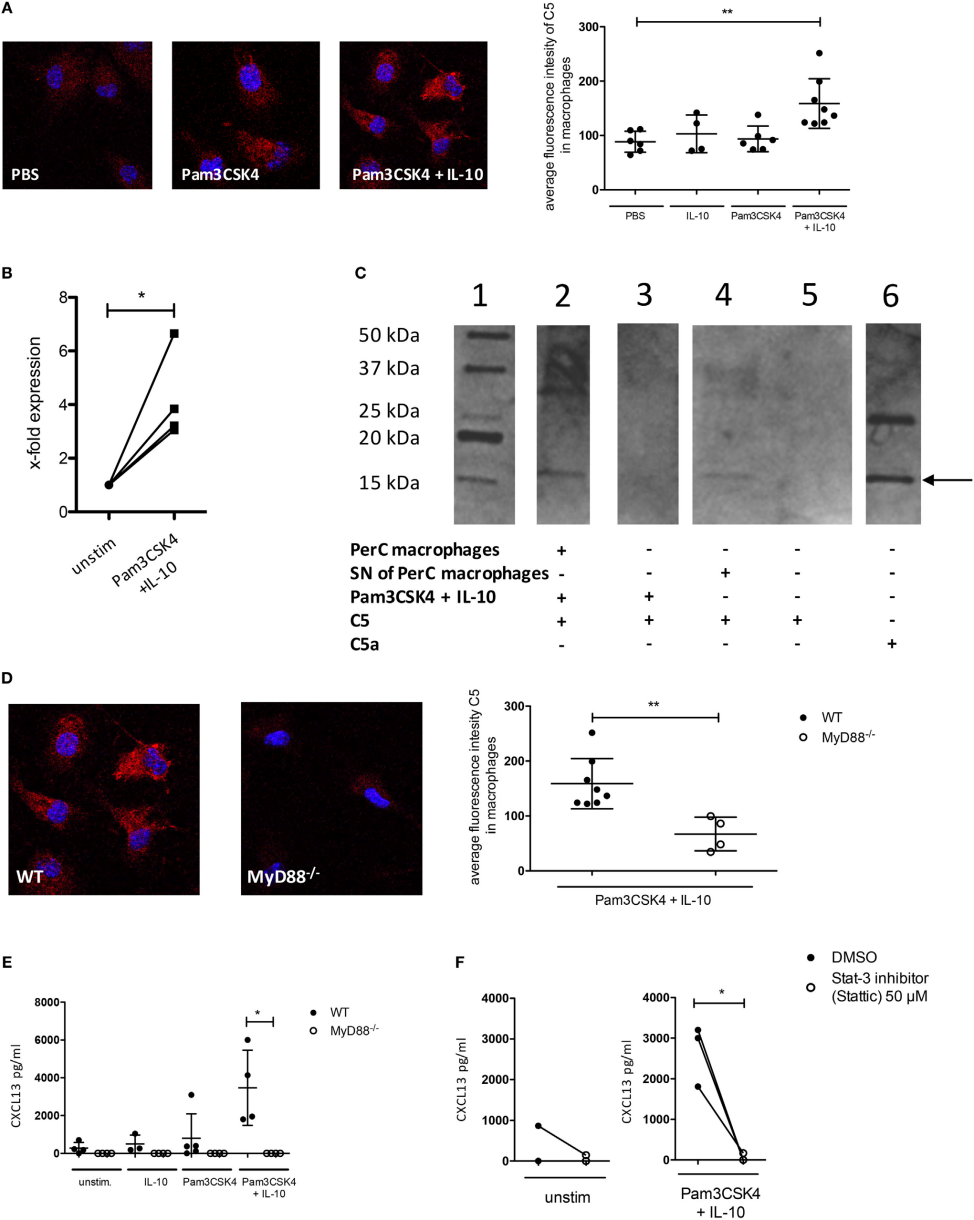
C5 production and non-canonical C5a generation in peritoneal macrophages following IL-10 and Pam3CSK4 stimulation. **(A)** Immunohistochemical analysis of C5 production from wild-type (wt) peritoneal macrophages (C57BL/6J background) in response to 24 h PBS, Pam3CSK4 or combined Pam3CSK4 + IL-10 stimulation (three pictures on the left). Quantification of C5 production based on the immunohistochemical analysis of C5 production (right panel). **(B)** Real-time RT-PCR for C5 using wt peritoneal macrophages (C57BL/6J background) 24 h after *in vitro* Pam3CSK4 + IL-10 stimulation compared to unstimulated controls. **(C)** Western Blot to determine C5 cleavage and C5a generation from wt peritoneal macrophages (C57BL/6J background) or their supernatant (SN) 24 h after *in vitro* Pam3CSK4 + IL-10 stimulation. Lane 1 = marker; lane 2 = Pam3CSK4 + IL-10-stimulated peritoneal macrophages in the presence of exogenous hC5; lane 3 = as in lane 2 but without cells; lane 4 = SN of Pam3CSK4 + IL-10-activated peritoneal macrophages incubated 4 h with exogenous hC5; lane 5 = as in lane 4 but without SN; lane 6 = hC5a purified from serum. The arrow depicts the monomeric C5a band. **(D)** Impact of MyD88-dependent cell signaling on Pam3CKS4 + IL-10-induced C5 production from peritoneal macrophages. The immunohistochemical analysis of C5 production from wt and MyD88^−/−^ peritoneal macrophages (both C57BL/6J background) is shown on the left. On the right, the quantification of the C5 production based on the evaluation of the immunohistochemical staining is shown. **(E)** CXCL13 production from unstimulated MyD88^−/−^ adherent PerC cells as well as after stimulation with IL-10, Pam3CSK4, or both compared to wt cells (both C57BL/6J background) and **(F)** from unstimulated (left panel) and Pam3CSK4 + IL-10-stimulated (right panel) wt adherent PerC cells in the presence or absence of the Stat-3 inhibitor Stattic (50 µM). Values shown in **(A,B,D,E)** are the mean ± SD. Statistical differences between unstimulated and stimulated samples or between wt and knockout cells were determined by Kruskal–Wallis test with Dunn’s *post hoc* test **(A,E)**, paired *t*-test **(B,F)**, or Mann–Whitney test **(D)**. *p < 0.05, **p < 0.01.

**Figure 5:**
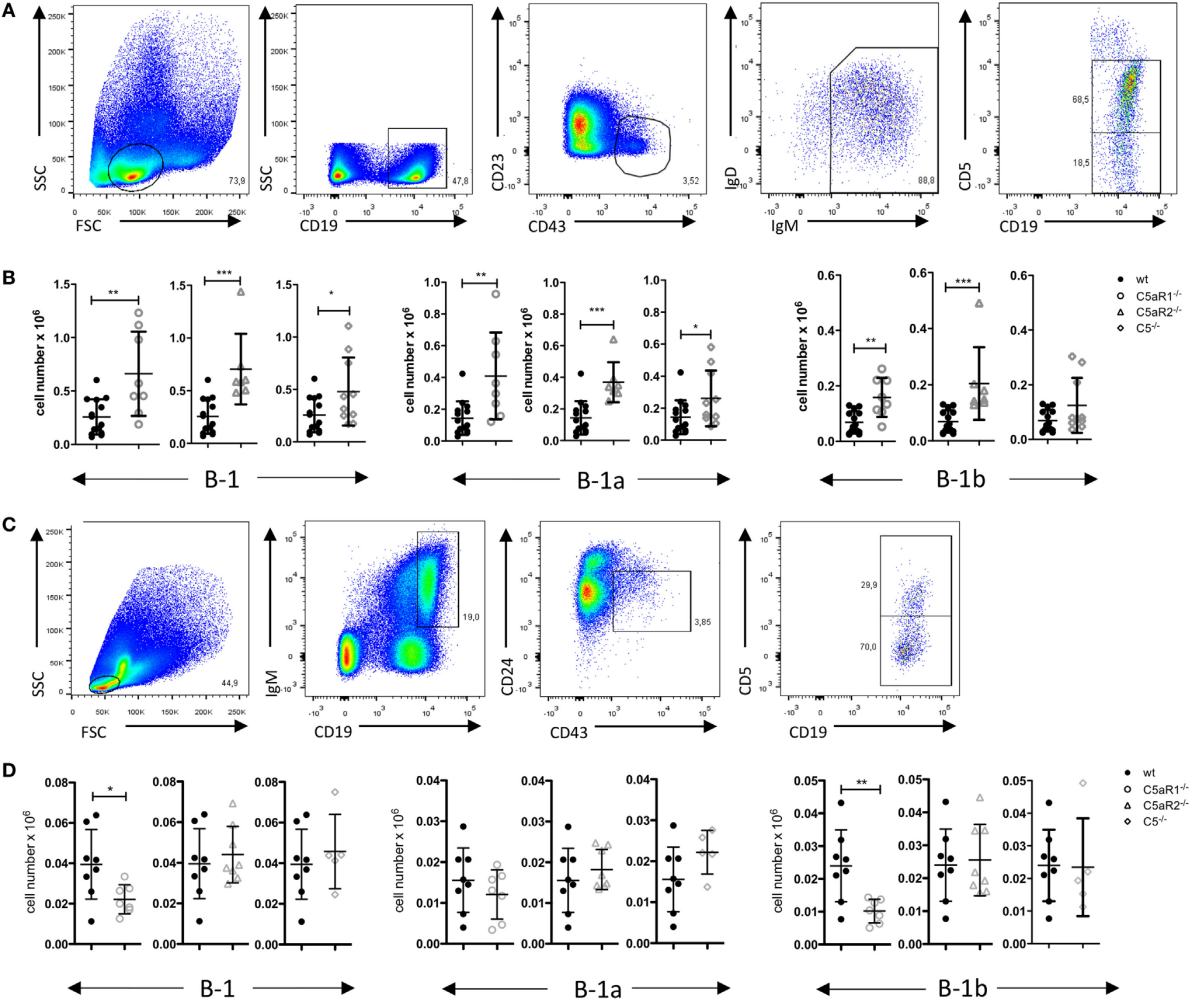
B-1 cell numbers in the spleen and bone marrow (BM) of wild-type (wt), C5aR1^−/−^, C5aR2^−/−^, and C5^−/−^ mice. **(A)** Gating strategy to identify total B-1, B-1a, and B-1b cells in the spleen. **(B)** Total B-1, B-1a, and B-1b cell numbers in the spleen of wt, C5aR1^−/−^, C5aR2^−/−^, and C5^−/−^ mice (all on C57BL/6J background). **(C)** Gating strategy to identify total B-1, B-1a, and B-1b cells in the BM. **(D)** Total B-1, B-1a, and B-1b cell numbers in the BM of wt, C5aR1^−/−^, C5aR2^−/−^, and C5^−/−^ mice. Values shown in **(B)** and **(D)** are the mean ± SD. Statistical differences between wt and knockout mice were determined by Mann–Whitney test **(B)** or unpaired *t*-test **(D)**. *p < 0.05, **p < 0.01, ***p < 0.001.

**Figure 6:**
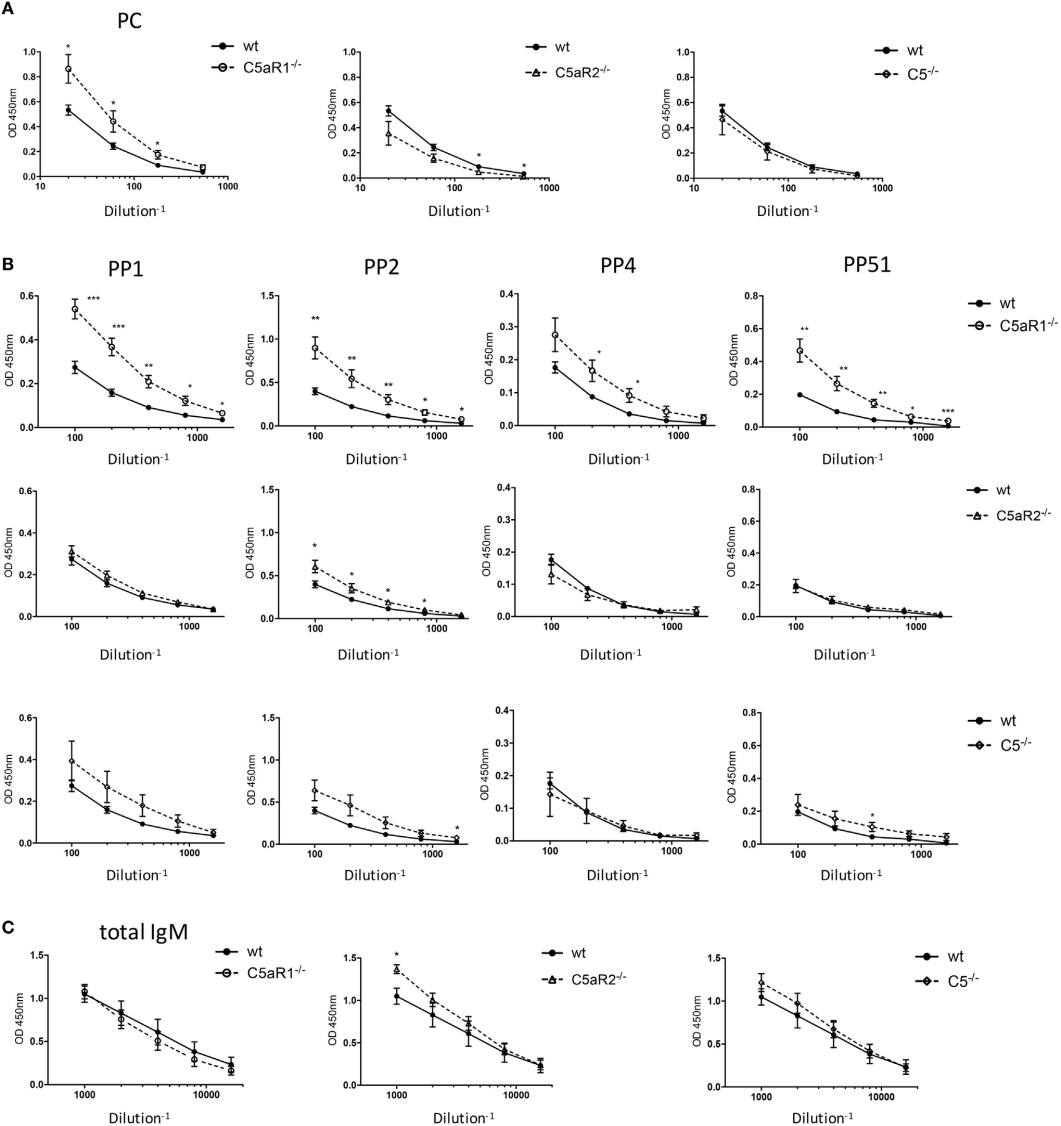
Total IgM and antigen-specific natural IgM (nIgM) serum titers in naïve wild-type (wt), C5aR1^−/−^, C5aR2^−/−^, and C5^−/−^ mice. **(A)** Serum titers of aPC-nIgM antibodies in wt, C5aR1^−/−^, C5aR2^−/−^, and C5^−/−^ mice (all on C57BL/6J background). Serum dilutions were 1:20, 1:60, 1:180, and 1:540. **(B)** Serum titers of anti-PP1, 2, 4, and 51 nIgM antibodies in wt, C5aR1^−/−^, C5aR2^−/−^, and C5^−/−^ mice. Serum dilutions were 1:100, 1:200, 1:400, 1:800, and 1:1,600 for anti-pneumococcal polysaccharides nIgM. **(C)** Serum titers of total IgM antibodies in wt, C5aR1^−/−^, C5aR2^−/−^, and C5^−/−^ mice. Serum dilutions were 1:1,000, 1:2,000, 1:4,000, 1:8,000, and 1:16,000. Values shown are the mean ± SEM. Statistical differences between wt and knockout mice were determined by an unpaired *t*-test. *p < 0.05, **p < 0.01, ***p < 0.001.

Statistical analysis was performed using the GraphPad Prism version 5.0c (GraphPad Software, Inc., USA). Each dot depicted in the graphs represents one individual (donor) mouse. Normal distribution of data was tested using the D’Agostino and Pearson omnibus normality test. If two independent groups were not normally distributed and could not be transformed to a normal distribution by logarithmic transformation, we used the non-parametric Mann–Whitney test (Figures 2D, 3C, 4D and 5B; Figure S1 in Supplementary Material). If two independent, normally distributed groups were compared, we used an unpaired *t*-test (Figures 1B, 2A,B,E,I,J, 3B, 5D and 6). The paired *t*-test was used in case that one measurement variable and two nominal variables were compared (Figures 2C,F–H, 3D,E and 4B,F; Figure S3 in Supplementary Material). To assess differences between multiple groups, non-parametric one-way ANOVA on ranks (Kruskal–Wallis) test was used with Dunn’s *post hoc* evaluation (Figures 3A and 4A,E).

## Results

### The C5a/C5aR Axes Control the Steady-State Dynamics of Peritoneal B-1a Cells

Germ-free mice have increased numbers of B-1 cells in their PerC as compared with SPF-housed mice, suggesting that microbial signatures control B-1 cell homeostasis in the peritoneum (23). Microbial patterns are strong activators of the complement system, which plays an important role in early defense against microbial invaders. In line with this view, we assessed whether complement activation regulates the dynamics of B-1 cells under steady-state conditions. For this purpose, we determined the numbers and composition of B-1 cells in the PerC of wt, C5aR1^−/−^, C5aR2^−/−^, and C5^−/−^, mice. After excluding CD11b^hi^ macrophages and CD5^hi^ T cells, we identified peritoneal B-1 cells as CD43^+^ and IgM^+^ cells. Among these cells, CD5^+^ and CD5^–^ cells were considered as B-1a and B-1b cells, respectively (Figure 1A). In C5aR1^−/−^ and C5aR2^−/−^, but not in C5^−/−^ mice, we found a significant reduction of total B-1 cell numbers under steady-state conditions as compared with wt mice. The reduction of total B-1 cells resulted mainly from a decrease in B-1a cell numbers, whereas B-1b cell numbers were in the same range (Figure 1B). Our findings suggest that the activation of the complement system, generation of C5a, and activation of the C5a/C5aR axes control the dynamics of peritoneal B-1a cells under steady-state conditions.

### The C5a/C5aR1 Axis Drives CXCL13 Production in the PerC under Steady-State Conditions

Previously, it has been demonstrated that CXCL13 production from cells in the omentum and from peritoneal macrophages is critical for B-1 cell homing into the peritoneum (21). To assess a potential impact of the C5a/C5aR axes on peritoneal CXCL13 production, we determined CXCL13 concentrations under steady-state conditions in the peritoneal lavage fluid from C5aR1^−/−^, C5aR2^−/−^, and C5^−/−^ mice and compared them to those of wt mice. In mice lacking C5aR1 or C5, we found significantly reduced levels of CXCL13 in the PerC under steady-state conditions as compared with wt mice. The knock-out of C5aR2 also resulted in a slight reduction of CXCL13; however, this reduction did not reach the level of statistical significance (Figure 2A). The same phenomenon was observed using wt and C5aR1- and C5aR2-deficient mice on the BALB/c background (Figure 2B). CXCL12 levels in the peritoneal lavage fluid were in the range of the detection limit of the assay (data not shown).

### The TLR2 Ligand Pam3CSK4 and IL-10 Drive CXCL13 Production from Peritoneal Macrophages through a C5-Dependent Mechanism

Previous findings demonstrated that stimulation of human dendritic cells with either a TLR ligand or IL-10 resulted in CXCL13 production, which was enhanced in the presence of both ligands (42). As PerC macrophages are an important source of intraperitoneal CXCL13 (21), we determined the production of CXCL13 by adherent PerC cells, i.e., mainly macrophages, *ex vivo* after stimulation with the TLR2 ligand Pam3CSK4 and/or IL-10. We selected IL-10 as it is a major cytokine produced by PerC B-1 cells (43) and Pam3CSK4 as a prototypic bacterial pathogen-associated molecular pattern. We found that Pam3CSK4 and IL-10 induced CXCL13 production from wt adherent PerC cells, which was markedly enhanced when both molecules were administered simultaneoulsy (Figure 2C). Non-adherent PerC cells from wt mice, comprising mainly B and T cells, which were stimulated with Pam3CSK4 did not secrete any detectable CXCL13 (Figure S1 in Supplementary Material), suggesting that it is the dominant fraction of macrophages within the adherent PerC cells that produces CXCL13 in response to Pam3CSK4 and/or IL-10 stimulation. Importantly, CXCL13 production from adherent PerC cells, which was minor without stimulation (Figure S2 in Supplementary Material), was significantly reduced when we stimulated C5aR1-, C5aR2, or C5-deficient cells with Pam3CSK4 and IL-10 (Figure 2D). These findings suggest that C5a drives CXCL13 production from adherent PerC macrophages through activation of C5aR1 and C5aR2. To test the relevance of this pathway *in vivo*, we injected C5a into the PerC of wt mice. Indeed, we found significantly increased peritoneal CXCL13 levels 6 h after C5a administration *in vivo* (Figure 2E). To better mimic the steady-state situation in the PerC, we stimulated total PerC cells with Pam3CSK4. As expected, we found significant CXCL13 production (Figure 2F; left panel). When we targeted the IL-10R with a blocking Ab, we observed a significant reduction of the CXCL13 production (Figure 2F; right panel), strongly suggesting that intrinsically produced B-1 cell-derived IL-10 synergizes with Pam3CSK4 to drive CXCL13 production from peritoneal macrophages. In contrast, the IgG isotype control Ab had no inhibitory effect (Figure S3A in Supplementary Material). Also, Pam3CSK4-mediated IL-10 production from total PerC cells was not affected by IL-10R blockage or isotype control Ab treatment (Figure 2G; Figure S3B in Supplementary Material). Furthermore, we assessed the impact of C5aR1/2 signaling on combined Pam3CSK4 and IL-10-driven CXCL13 production by pharmacological targeting using the C5aR antagonist A8^Δ71–73^ (C5aRA), which targets C5aR1 and C5aR2. Simultaneous blockade of C5aR1 and C5aR2, which had no effect on unstimulated adherent PerC cells, significantly reduced Pam3CSK4 + IL-10-induced CXCL13 production from such cells (Figure 2H). In line with these findings, we found reduced peritoneal CXCL13 levels (Figure 2I) as well as reduced total B-1, B-1a, and B-1b cell numbers (Figure 2J) in the PerC of TLR2^−/−^ mice as compared with wt mice under steady-state conditions. These data suggest that synergistic TLR2 and IL-10 receptor activation drive C5 production and generation, which in turn activates C5aR1 and C5aR2 to produce CXCL13 from peritoneal macrophages.

### TLR2 Activation Drives IL-10 Production by B-1a Cells Independent of the C5a/C5aR Axes

Since IL-10 is an important co-stimulus to drive CXCL13 production by peritoneal macrophages, we next assessed, if altered IL-10 production from peritoneal cells may account for the reduced CXCL13 levels that we observed in response to combined Pam3CSK4 and IL-10 stimulation of adherent PerC in mice deficient for C5, C5aR1, or C5aR2. In a first step, we confirmed non-adherent PerC cells as the main IL-10 source after Pam3CSK4 stimulation (Figure 3A). To identify the IL-10-producing cell type within the group of non-adherent PerC cells, we isolated peritoneal cells from IL-10 GFP-reporter mice (VertX mice) and determined their GFP expression under steady-state conditions. Under these conditions, we observed GFP (IL-10) expression mainly in B-1a cells and to a minor extent in B-1b cells, but not in B-2 cells (Figure 3B) or other cell types including T cells (data not shown). In B-cell-specific IL-10 knockouts (IL-10^fl^CD19^cre^), total B-1 cells and more specifically B-1a but not B-1b cells were significantly reduced as compared with litter mate control mice (IL-10^fl^CD19^wt^), suggesting that B-1a cell-derived IL-10 is necessary for their homeostasis in the PerC (Figure 3C). Finally, we determined the impact of C5aR1-, C5aR2-, and C5-deficiency on TLR2-driven IL-10 production from B-1 cells. We found a strong but similar upregulation of IL-10 in non-adherent PerC cells from all mouse strains (Figure 3D). Further, blocking the C5aR axes by the C5aRA had no impact on Pam3CSK4-induced IL-10 production (Figure 3E). These data suggest that the reduced CXCL13 production in response to combined IL-10 receptor/TLR2 activation in C5aR1-, C5aR2-, and C5-deficient mice (Figure 2D) is not regulated at the level of B-1 cells. In support of this view, we found at best a very minor expression of C5aR1 in peritoneal B-1 cells using GFP-C5aR1 knock-in mice or staining for C5aR1 surface expression (Figure 3F).

### TLR2 and IL-10 Receptor Activation Synergize to Induce C5 Production and Subsequent Generation of C5a by Peritoneal Macrophages

As the C5a/C5aR axes did not regulate IL-10 release from B-1 cells, we hypothesized that CXCL13 production from peritoneal macrophages is regulated through paracrine C5 production in response to combined IL-10 and TLR2 stimulation. We determined C5 production in peritoneal macrophages after stimulation with Pam3CSK4 and/or IL-10 using confocal microscopy. We found a significant induction of intracellular C5 only when Pam3CSK4 and IL-10 were administered together (Figure 4A). The strong upregulation of C5 after stimulation, which we observed by confocal microscopy was confirmed by real-time RT-PCR in peritoneal macrophages, where we found a fourfold upregulation of C5 expression 24 h after stimulation with Pam3CSK4 + IL-10 (Figure 4B). In a next step, we incubated Pam3CSK4 + IL-10-stimulated wt macrophages (using DMEM medium without FBS) or SNs of such macrophages with C5 as previously described (32). We found C5 cleavage into C5a, suggesting the presence of a soluble protease expressed by the stimulated macrophages (Figure 4C). To test whether the activation of MyD88 downstream of TLR2 and activation of IL-10 receptor is critical for C5 production, we used MyD88-deficient mice or blocked IL-10 receptor signaling using the Stat-3 inhibitor Stattic. Both, intracellular production of C5 as well as CXCL13 release by peritoneal macrophages were dependent on MyD88 signaling (Figures 4D,E). CXCL13 production was additionally dependent on Stat-3 signaling (Figure 4F). Thus, combined IL-10R and TLR2 stimulation of peritoneal macrophages drives paracrine C5 production and non-canonical generation of C5a by a secreted, cell-specific protease.

### C5aR Deficiency Leads to Increased B-1 Cell Numbers in the Spleen

To assess whether the observed reduction of B-1a cells in the PerC is associated with changes of B-1 cells in other compartments, we determined B-1 cell numbers in the spleen and BM. The gating strategy for B-1 cells and their subsets in spleen and BM was adapted from Yenson and Baumgarth (44). In the spleen, we characterized B-1 cells as CD19^+^, CD43^+^, CD23^–^, IgM^+^, IgD^lo^, CD5^+/−^ (Figure 5A). In contrast to the decreased B-1 cell numbers in the PerC, we found increased numbers of total splenic B-1 cells in C5aR1^−/−^, C5aR2^−/−^, and C5^−/−^ mice (Figure 5B). This increase resulted from a higher number of the B-1a cell subpopulation (Figure 5B). In contrast to our observation in the peritoneum, C5aR1 and C5aR2 deficiency did also affect splenic B-1b cell numbers, which increased similar to B-1a cells (Figure 5B). Additionally, we determined the impact of the C5a/C5aR axes on B-1 cell numbers in the BM. We characterized B-1 cells as CD19^+^, IgM^+^, CD43^+^, CD24^−^, CD5^+/−^ cells (Figure 5C). We observed a significant reduction of total B-1 cells in C5aR1^−/−^ mice, whereas they were not affected by C5aR2 or C5 deficiency (Figure 5D). The drop in B-1 cells in C5aR1-deficient mice was mainly due to a reduced number of B-1b cells. Taken together, our findings suggest that the activation of the complement system, generation of C5a, and activation of the C5a/C5aR axes control the dynamics of B-1a cells under steady-state conditions, i.e., the migration between PerC, spleen, and BM. Further, our data imply a synergistic role for C5aR1 and C5aR2 in the control of B-1a cell homing into the peritoneum and B-1a and B-1b cell homing into the spleen. In contrast, C5aR1, but not C5aR2, activation appears to be critical for the presence of B-1b cells in the BM.

### C5aR1-Deficient Mice Show Increased Serum Levels of nIgM Antibodies Specific for Phosphorylcholine and PP

An important function of B-1 cells in the spleen and the BM is the secretion of nIgM antibodies (7), which not only serve as a first shield against invading pathogens but play important roles in tissue homeostasis. As we found significantly increased B-1 cells in the spleen of C5aR1^−/−^ mice, we next determined whether this increase in B-1 cell numbers is associated with increased nIgM antibody titers in the circulation. Indeed, C5aR1^−/−^ mice had markedly elevated levels of nIgM antibodies specific for PC (Figure 6A) or PP of serotypes 1, 2, 4, and 51 (Figure 6B, upper row) as compared with wt control sera, whereas the total serum IgM titers in the sera of wt and C5aR1^−/−^ mice were similar (Figure 6C, left graph). Further, we found slightly increased nIgM antibody titers against PP1, PP2, and PP51 in the sera of C5^−/−^ mice and PP2 in the sera of C5aR2^−/−^ mice (Figure 6B, middle and lower row). Total IgM serum titers in the sera of C5aR2^−/−^ and C5^−/−^ mice were only slightly increased (Figure 6C, middle and right graph).

## Discussion

B-1 cells comprise a heterogeneous population of cells that reside in body cavities, BM, spleen, and skin. They develop in several waves first from yolk sac and paraaortic splanchnopleura, followed by fetal BM and liver and eventually after birth from the BM (45). Out of the B-1 cell pool, non-terminally differentiated B-1a and B-1b cells within the spleen and the BM, but not the PerC, produce nIgM antibodies. At this point, it is unclear whether B-1 cells from the PerC or the BM migrate to the spleen during homeostatic conditions to produce nIgM antibodies or a constant pool of nIgM-producing B-1 cells resides within the BM and the spleen that is replenished by self-renewal. We found that complement and, in particular, the C5a/C5aR axes have a significant impact on B-1a cell numbers in the PerC and the spleen and on B-1b numbers in the spleen and BM. The reduced number of PerC B-1a cells and the concurrent increase in splenic B-1a cells in C5aR1^−/−^ and C5aR2^−/−^ mice suggests that C5a generation and activation of these receptors regulates the trafficking between the two compartments and/or self-renewal of B-1a cells in opposing ways. In contrast to the initial view that C5aR2 solely acts as a decoy receptor simply counteracting C5aR1-mediated effector functions, recent results show that C5aR2 exerts pro- as well anti-inflammatory effects either in concert with or independent of C5aR1 (46–48). In CLP-driven sepsis, for example, only the combined inhibition of C5aR1 and C5aR2 improves animal survival (49). Our findings that C5aR1 and C5aR2 control peritoneal and splenic B-1 cell numbers adds to the growing body of data demonstrating joint regulatory functions. Strikingly, we also observed a marked increase of B-1b cells in the spleen of C5aR1^−/−^ mice that was associated with a decrease in BM B-1b cells, which may indicate that the C5a/C5aR1 axis controls the egress of B-1b cells from the BM into the spleen under steady-state conditions. In support of this view, we found a substantial increase of nIgM antibody production in C5aR1^−/−^ mice recognizing PC and several *S. pneumoniae*-derived polysaccharides. Indeed, the available data from the literature suggest that splenic plasma cells from B-1a and B-1b cells contribute to nIgM production (50).

CXCL13 produced by peritoneal macrophages and the omentum drives B-1 cell homing into the PerC (21). In line with the data generated by Perrier et al. (42), we found that IL-10 induces some CXCL13 production from peritoneal macrophages that can be markedly enhanced by additional stimulation with the TLR2-ligand Pam3CSK4. Data obtained with germ-free mice suggest that leakage of TLR ligands from commensal intestinal microbiota into the PerC is critical for peritoneal CXCL13 production, as the number of B-1 cells is much higher in germ-free mice than in mice housed under SPF (23). Using IL-10 reporter mice, we confirmed that B-1 and, in particular, B-1a cells are the main source of IL-10 in the PerC (43). Our data further confirmed that such IL-10 is of major importance for B-1a cells, as the number of PerC B-1 cells was significantly reduced in B cell-specific IL-10-deficient mice. Interestingly, C5 and the C5a/C5aR axes were not involved in TLR2-driven IL-10 production, but had a strong impact on CXCL13 production under steady-state conditions. More specifically, the lack of C5aR1 was associated with a significant reduction of CXCL13 levels in the peritoneum under steady-state conditions. In line with this view, i.p. injection with C5a resulted in increased intraperitoneal CXCL13 levels. In search for mechanisms, we found that peritoneal macrophages need at least three signals to efficiently produce CXCL13, i.e., IL-10 receptor, TLR, and C5aR activation. Our findings suggest a model in which IL-10 receptor and TLR2 activation induce the production and the non-canonical cleavage of C5 into C5a in PerC macrophages by a cell-derived protease. Surprisingly, it is the combined interaction of C5a with C5aR1 and C5aR2 that is necessary for CXCL13 production. C5 production from extrahepatic sources has been described before in human alveolar macrophages in response to LPS stimulation (51) and in murine thioglycollate-elicited macrophages (52). Further, alveolar macrophages from rats were shown to cleave C5 into C5a by a cell-specific protease (32). Our data that non-canonical C5a generation and C5aR activation regulates chemokine production from peritoneal macrophages adds to the growing body of evidence that local, cellular complement activation is of major importance for tissue homeostasis (53).

In summary, we uncovered an unexpected, novel role for complement in the regulation of the B-1 cell compartment that affects both the distribution of B-1a and B-1b B cells in the PerC, spleen, and BM during steady-state and the production of nIgM antibodies. We describe a novel mechanism, by which TLR2-driven IL-10 production from B-1a cells synergizes with TLR2-driven activation of PerC macrophages to induce non-canonical C5a production as an important signal to promote CXCL13 production from such macrophages. Since their initial description in the 1990s, a body of evidence has accumulated that B-1 cells play important roles in the inflammatory response to infection, autoimmunity, ischemia/reperfusion injury, obesity, and atherosclerosis (54). Remarkably, this list of inflammatory entities has a strong overlap with diseases that are regulated by complement (26) suggesting an unanticipated link between B-1 cell/complement interactions that awaits further exploration.

## Ethics Statement

All animal studies were reviewed and approved [number: V242—81505/2016 (19-2/17)] by local authorities of the Animal Care and Use Committee (Ministerium für Energiewende, Landwirtschaft, Umwelt, Natur und Digitalisierung, Kiel, Germany) and performed by certified personnel.

## Author Contributions

KB, JF, and AM conducted key studies and analyzed the data. RM provided VertX mice. CK and JK designed and coordinated the study, analyzed the data and wrote the manuscript.

## Conflict of Interest Statement

The authors declare that the research was conducted in the absence of any commercial or financial relationships that could be construed as a potential conflict of interest.

## Supplementary Material

Figure S1 *In vitro* CXCL13 production by non-adherent cells from the peritoneal cavity from wild-type mice (C57BL/6J background) 24 h after stimulation with Pam3CSK4 (*n* = 5 per group). Values shown are the mean ± SD. Statistical differences between unstimulated and stimulated samples were determined using Mann–Whitney test.

Figure S2 *In vitro* CXCL13 production from adherent peritoneal cavity cells of wild-type, C5aR1-, C5aR2-, and C5-deficient mice (all C57BL6/J background) after 24 h in culture without stimulation. Values shown are the mean ± SD.

Figure S3 *In vitro* **(A)** CXCL13 and **(B)** IL-10 production from total PerC cells from wild-type mice 24 h after stimulation with Pam3CSK4 compared to Pam3CSK4 + isotype control. Statistical differences were determined using a paired *t*-test.
