# Supplementary figures and images for: A Novel Role for C5a in B-1 Cell Homeostasis
Source: Front Immunol. 2018 Feb 19;9:258. doi: 10.3389/fimmu.2018.00258 (PMC5827565; doi:10.3389/fimmu.2018.00258)

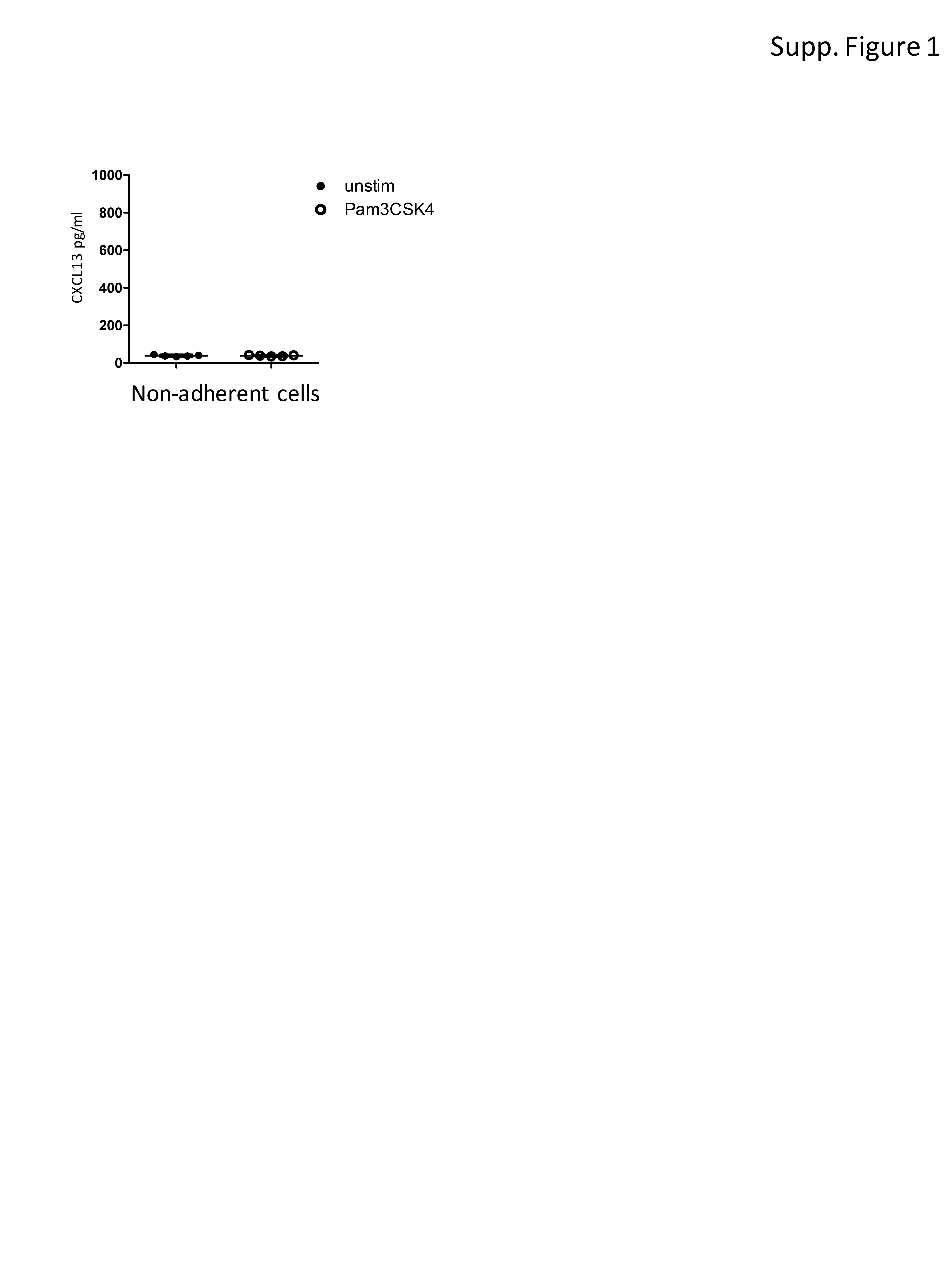

Supplement: Figure S1 — In vitro CXCL13 production by non-adherent cells from the peritoneal cavity from wild-type mice (C57BL/6J background) 24 h after stimulation with Pam3CSK4 (n = 5 per group). Values shown are the mean ± SD. Statistical differences between unstimulated and stimulated samples were determined using Mann–Whitney test. [file Image_1.jpeg]

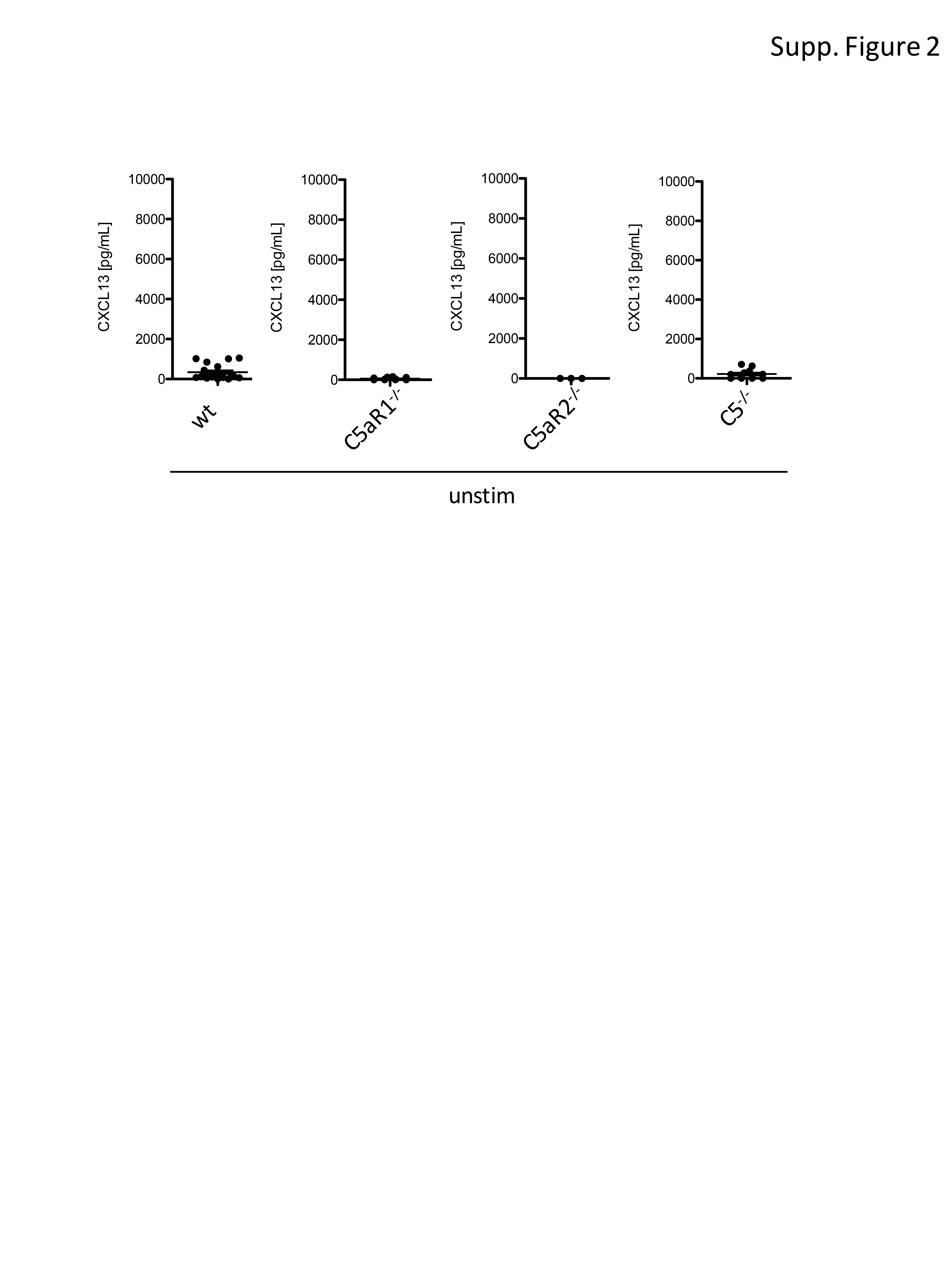

Supplement: Figure S2 — In vitro CXCL13 production from adherent peritoneal cavity cells of wild-type, C5aR1-, C5aR2-, and C5-deficient mice (all C57BL6/J background) after 24 h in culture without stimulation. Values shown are the mean ± SD. [file Image_2.jpeg]

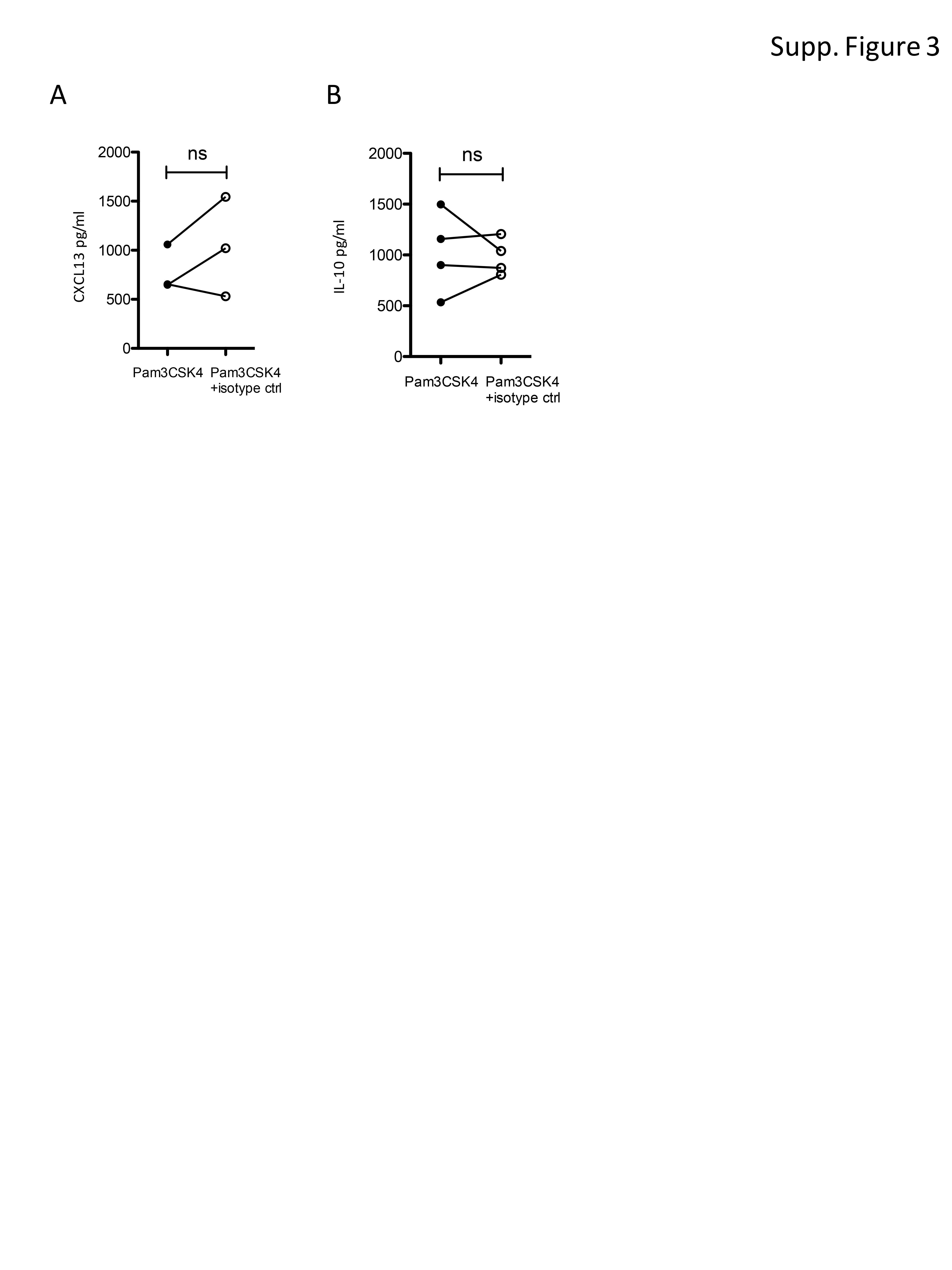

Supplement: Figure S3 — In vitro (A) CXCL13 and (B) IL-10 production from total PerC cells from wild-type mice 24 h after stimulation with Pam3CSK4 compared to Pam3CSK4 + isotype control. Statistical differences were determined using a paired t-test. [file Image_3.jpeg]
